# Supplementary material for: High-throughput deconvolution of 3D organoid dynamics at cellular resolution for cancer pharmacology with Cellos
Source: Nat Commun. 2023 Dec 18;14:8406. doi: 10.1038/s41467-023-44162-6 (PMC10730814; doi:10.1038/s41467-023-44162-6)
Supplement: Supplementary file 1 — Supplementary Information [file 41467_2023_44162_MOESM1_ESM.pdf]

Supplementary Information

High-throughput deconvolution of 3D organoid dynamics at cellular resolution for cancer pharmacology with Cellos

Patience Mukashyaka[1,2#], Pooja Kumar[1#], David J. Mellert[1], Shadae Nicholas[1], Javad Noorbakhsh[1], Mattia Brugiolo[1], Elise T. Courtois[1], Olga Anczukow[1,2], Edison T. Liu[1*], Jeffrey H. Chuang[1,2*]

[#] *Equal contribution*

[1] *The Jackson Laboratory for Genomic Medicine, Farmington, CT, USA*
[2]*Department of Genetics and Genome Sciences, University of Connecticut Health Center, Farmington, CT, USA*

[*] *Corresponding authors*
Edison T. Liu: ed.liu@jax.org, Jeffrey H. Chuang: jeff.chuang@jax.org

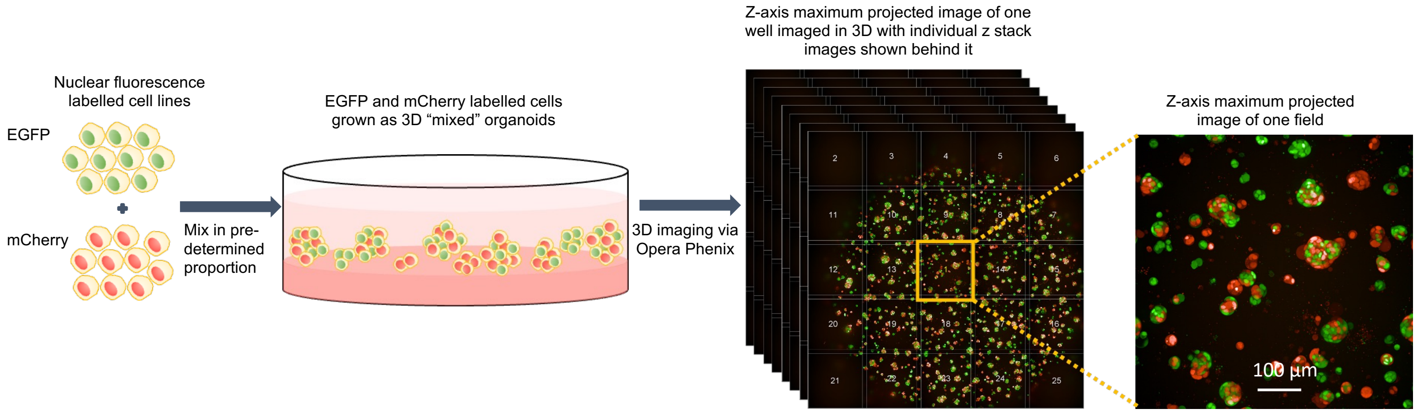

Supplementary Figure 1: Schematic representation of 3D organoid culture and imaging platform. EGFP and mCherry labeled cells are mixed in pre-determined proportion in culture to form 3D "mixed" organoids. The Opera Phenix system is then used to image the organoids in 3D. 25 fields are imaged per well.

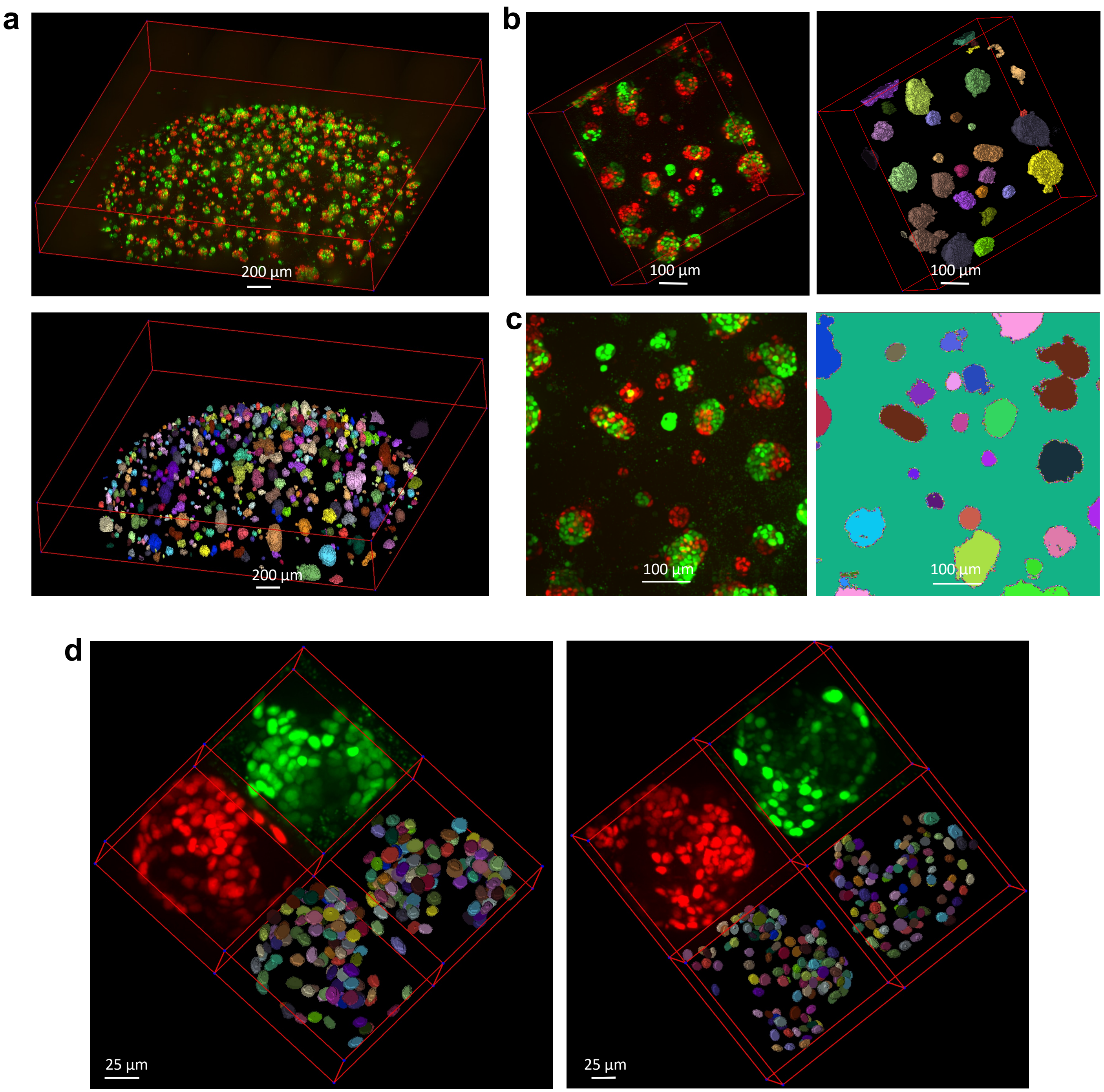

Supplementary Figure 2: Visualization of Cellos organoids and nuclei segmentation in drug free conditions. a. Top panel shows a 3D fluorescence image of one well with organoids consisting of A50 cells labeled with EGFP and B cells labeled with mCherry. Bottom panel shows organoids segmented by Cellos in 3D for the same well. Segmented organoids are indicated in different randomly selected colors. **b**. For better visualization, left and right panels show organoids and Cellos segmentation of organoids for one field in 3D. **c**. Representative z-axis maximum projected images of organoids (left panel) and Cellos organoid segmentation (right panel) for a field. **d**. Nuclei segmentation of EGFP and mCherry labeled nuclei. 3D grid visual representation of segmentation of EGFP and mCherry labeled nuclei with various fluorescence intensities. Separate example organoids are depicted in the two panels. Scale bar dimensions are as indicated.

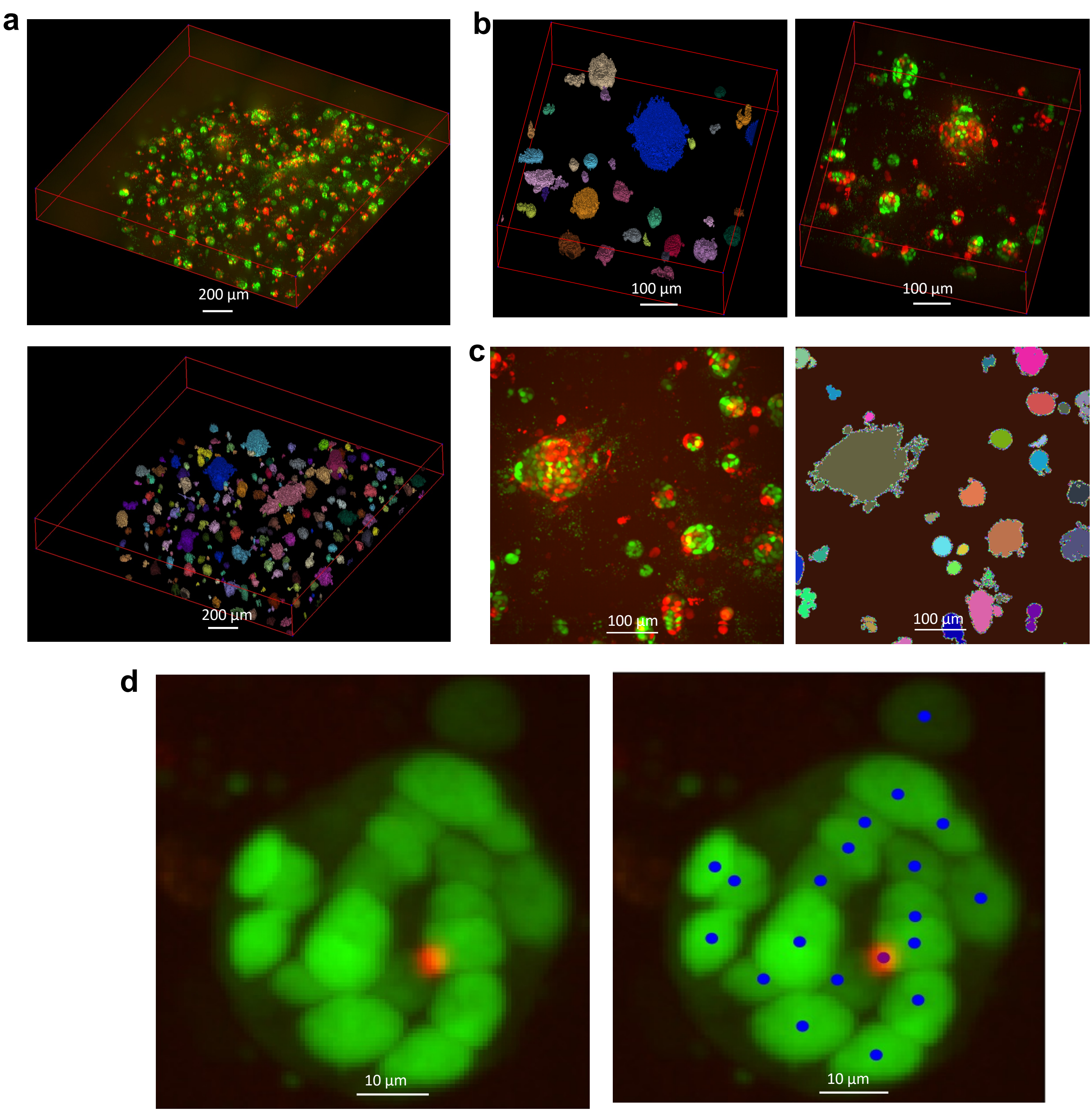

Supplementary Figure 3: Visualization of Cellos organoids and nuclei segmentation after 2 µM cisplatin treatment. a. Top panel shows a 3D fluorescence image of one well with organoids consisting of A50-EGFP and B-mCherry cells after 2 µM cisplatin treatment for four days. Bottom panel shows organoids segmented by Cellos in 3D of the whole well. Individual organoids and are in distinct colors in images after segmentation. **b**. For better visualization, left and right panels show organoids and Cellos segmentation of organoids for one field in 3D. **c**. Representative z-axis maximum projected images of organoids (left panel) and Cellos organoid segmentation (right panel) for a field post treatment. **d**. Left panel is an image of one organoid consisting of A50-EGFP and B-mCherry cells post treatment. Right panel shows the Cellos nuclear segmentation on the same organoid. Blue dots indicate A50-EGFP segmented nuclei and the purple dot marks B-mCherry segmented nucleus. Scale bar dimensions are as indicated.

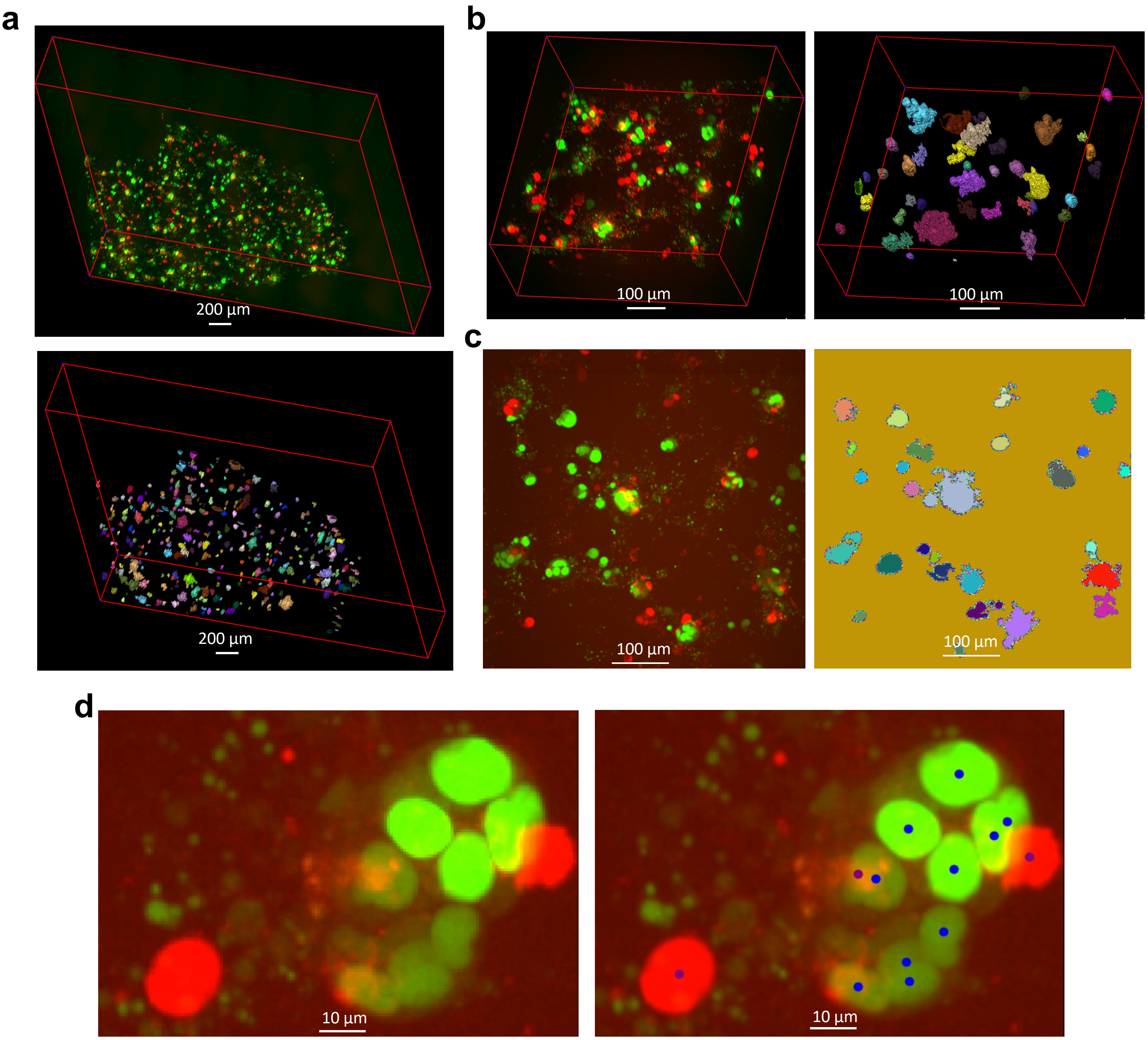

Supplementary Figure 4: Visualization of Cellos organoids and nuclei segmentation after 16 µM cisplatin treatment. a. Top panel shows a 3D fluorescence image of one well with organoids consisting of A50-EGFP and B-mCherry cells after 16 µM cisplatin treatment for four days. Bottom panel shows organoids segmented by Cellos in 3D of the whole well. Individual organoids and are in distinct colors in images after segmentation. **b**. For better visualization, left and right panels show organoids and Cellos segmentation of organoids for one field in 3D. **c**. Representative z-axis maximum projected images of organoids (left panel) and Cellos organoid segmentation (right panel) for a field. **d**. Left panel is an image of a small field consisting of organoids with A50-EGFP and/or B-mCherry cells post treatment. Right panel shows the Cellos nuclear segmentation on the same field. Blue dots indicate A50-EGFP segmented nuclei and purple dots mark B-mCherry segmented nuclei. Scale bar dimensions are as indicated.

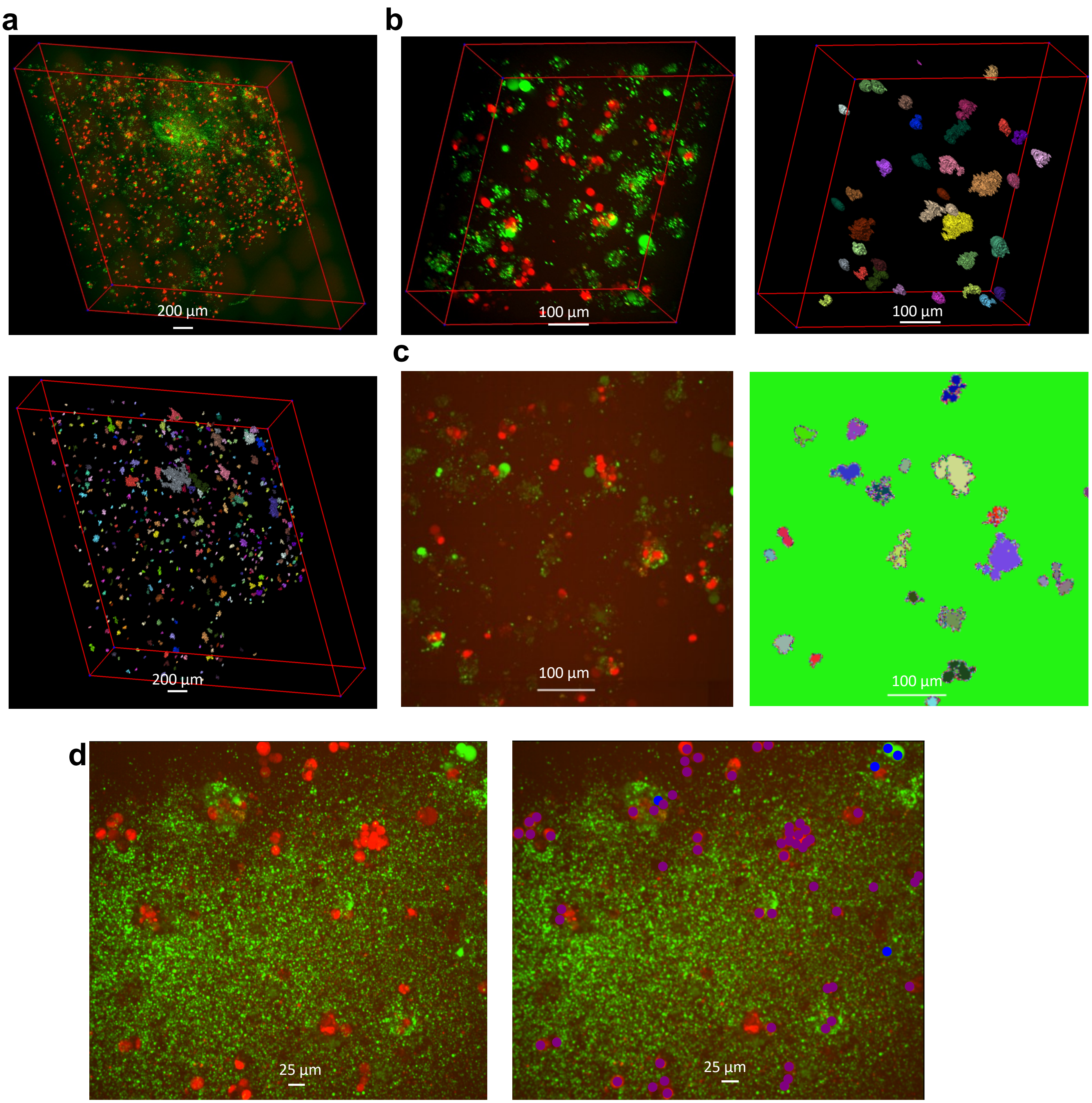

Supplementary Figure 5: Visualization of Cellos organoids and nuclei segmentation after 128μM cisplatin treatment. a. Top panel shows a 3D fluorescence image of one well with organoids consisting of A50-EGFP and B-mCherry cells after 128 μM cisplatin treatment for four days. Bottom panel shows organoids segmented by Cellos in 3D of the whole well. Individual organoids and are in distinct colors in images after segmentation. **b**. For better visualization, left and right panels show organoids and Cellos segmentation of organoids for a field in 3D. **c**. Representative z-axis maximum projected images of organoids (left panel) and Cellos organoid segmentation (right panel) for a field post treatment. **d**. Left panel is an image of a small field consisting of organoids with A50-EGFP and/or B-mCherry cells post treatment. Right panel shows the Cellos nuclear segmentation on the same field. Blue dots indicate A50-EGFP segmented nuclei and purple dots mark B-mCherry segmented nuclei. Scale bar dimensions are as indicated.

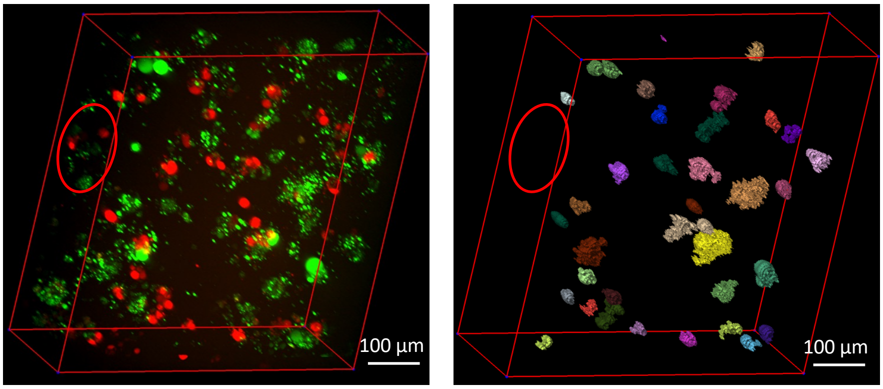

Supplementary Figure 6: Organoid segmentation limitations post high dose treatment. Image showing example of organoid segmentation (right panel) limitation after exposure to high concentration of drug (Cisplatin =128µM, left panel). Circled region shows single cells not segmented as organoids. Scale bar dimensions are as indicated.

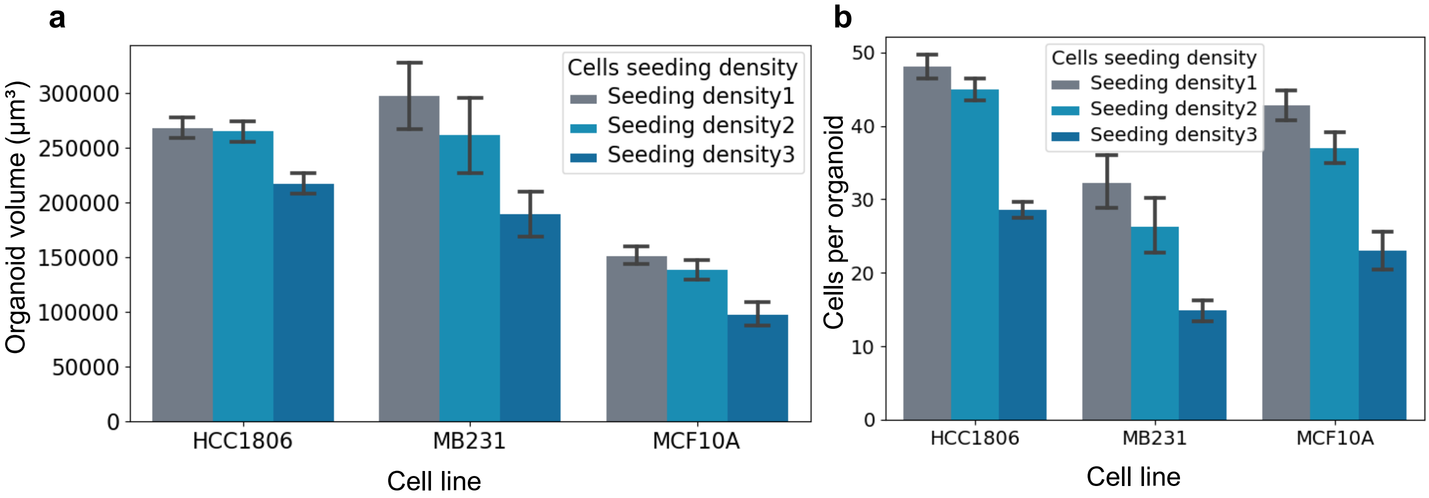

Supplementary Figure 7: Cellos analysis on diverse organoid morphologies. a. Cellos-measured organoid volumes for three different cell seeding densities (seeding density1 (high), seeding density2 (medium), and seeding density3 (low)), for each of the three cell lines. At least three replicate wells were analyzed for each condition. In total 11,416 organoids were analyzed and mean values for each condition are plotted with error bars representing the 95% confidence interval. **b.** Cellos-measured number of cells per organoid, for three decreasing cells seeding densities indicated as seeding density 1, 2, and 3 for each of the three cell lines. Mean values for each condition are plotted and error bars represent the 95% confidence interval and a total of 426,810 cells were examined. Source data for all plots are provided as source data files**.**

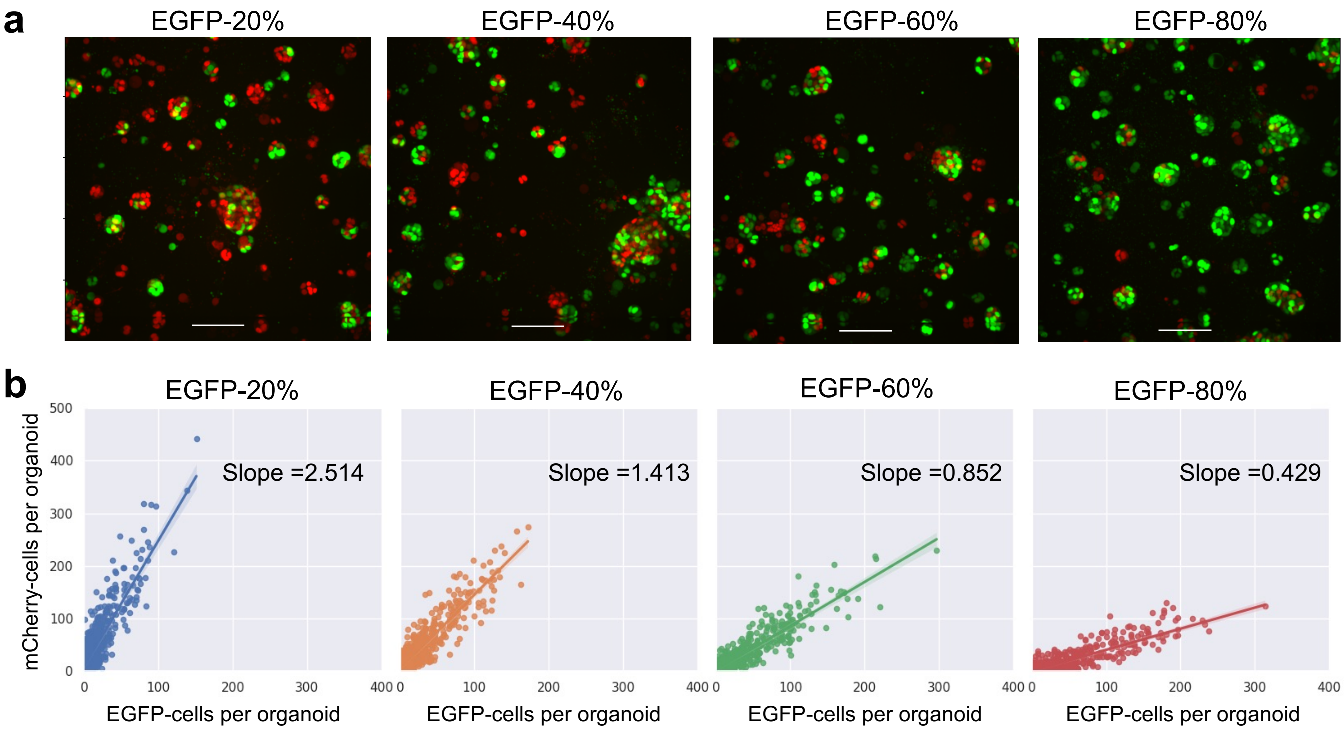

Supplementary Figure 8: Quantification of fluorescently labelled cell populations at the organoid resolution. a Representative z-axis maximum projection images of homogeneously mixed organoids generated with seeding percentages of 20%, 40%, 60% and 80% A50-EGFP, respectively, with the remaining cells being A50-mCherry. Images are from day0, and scale bar represents 100 µm. **b.** Number of EGFP vs mCherry cells detected in each homogeneously mixed B organoid. Each dot depicts an organoid. 3459 organoids with 166,543 cells were analyzed. Seeding conditions of EGFP-20% (blue), EGFP-40% (orange), EGFP-60% (green) and EGFP-80% (red) are shown from left to right. Slope of the fitted linear regression is noted with the shaded bands showing 95% confidence intervals. Source data for all plots are provided as source data files**.**

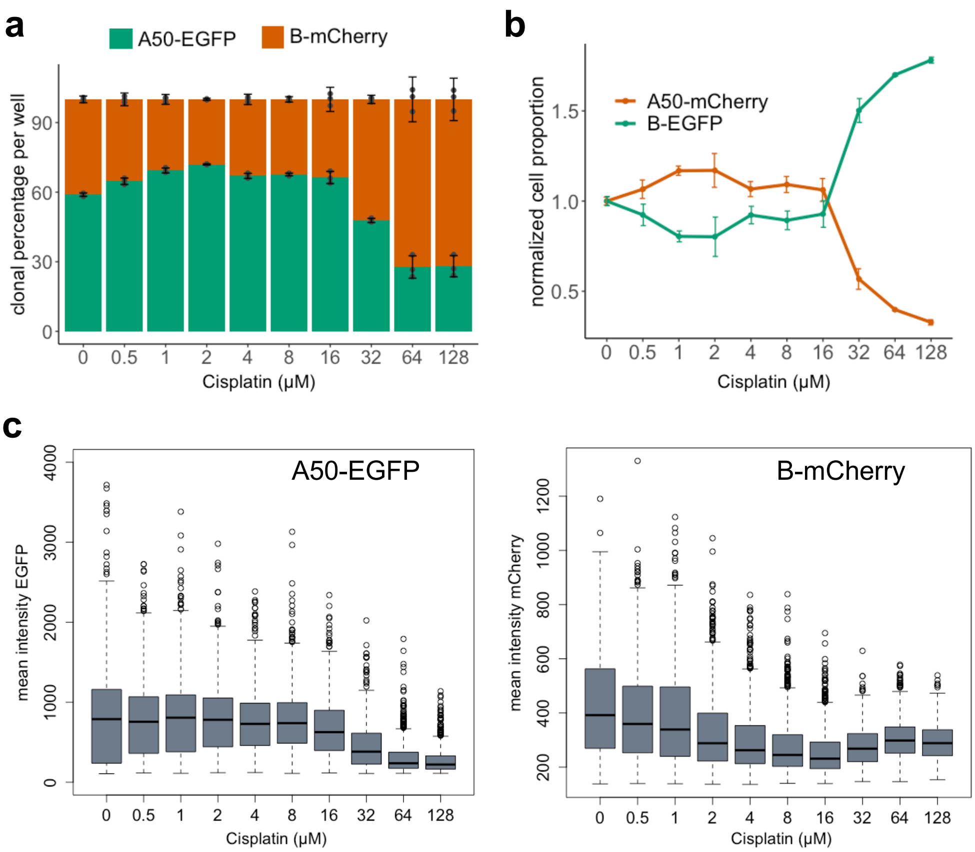

Supplementary Figure 9: Quantification of clonal response to cisplatin treatment. a. Percentage of A50-EGFP and B-mCherry cells detected by Cellos for increasing concentrations of cisplatin treatment. Each well was treated for 4 days. Values represent mean clonal percentages across three replicates, error bars show standard deviation and individual replicate data are shown as dots. A total of 137,765 cells across replicate conditions were assayed. **b**. Normalized cell proportions for A50-mCherry and B-EGFP clones when co-cultured as heterogeneously mixed organoids and treated with cisplatin for 4 days. Mean and standard deviation values of replicates for each condition are plotted. In total 100,312 cells were examined. For all conditions three replicate wells were analyzed, except one condition where two replicates were available. **c**. Mean intensity of EGFP (left panel) or mCherry (right panel) in heterogeneously mixed organoids consisting of A50-EGFP and B-mCherry. Organoids from three replicate wells are combined with a total of 8,382 organoids assayed. Horizontal line in the boxplot indicates the median, the box denotes the inter quartile range (IQR), the whiskers extend to a maximum of 1.5 times the IQR and outliers are shown as dots. Source data for all plots are provided as source data files.

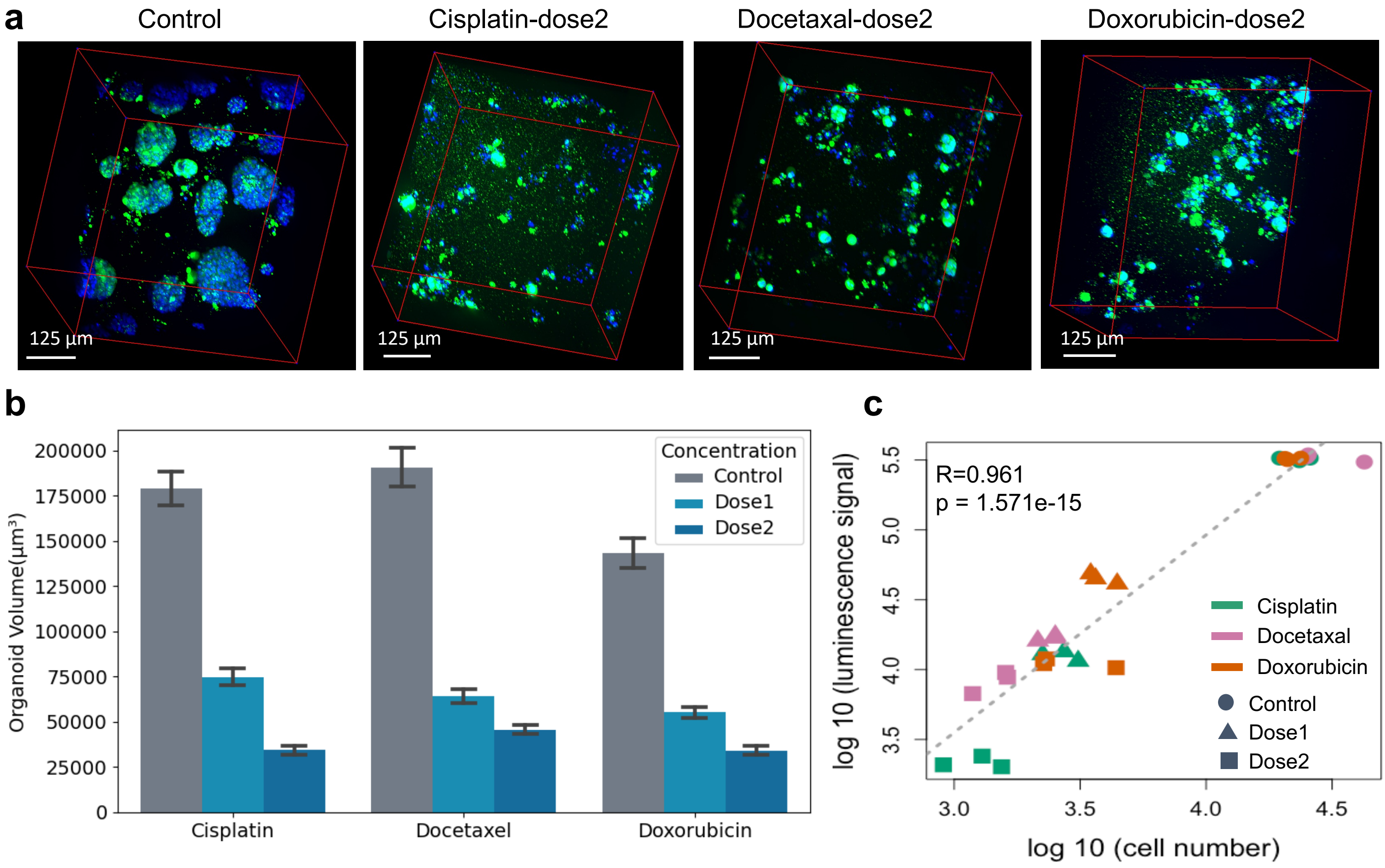

Supplementary Figure 10: Cellos analysis of organoids treated with different drugs. a. 3D images showing HCC1806 organoids treated with different drugs from left to right (control, cisplatin-dose2, Docetaxel-dose2, Doxorubicin-dose2). **b.** Barplot depicting organoid volume changes at different doses of the three drugs. Mean values per condition are plotted and the error bars show the 95% confidence intervals. A total of 14,250 organoids from three replicates for each condition were analyzed. **c.** Correlation plot of total number of cells counted by Cellos versus the luminescence signal for individual wells across different treatment conditions . Each datapoint represents the mean value for the well. A total of 271,011 segmented nuclei were included in this analysis. Drug treatments are shown as Cisplatin (green), Docetaxal (pink) and Doxorubicicn (orange) and and dosage conditions are as indicated in legend. Pearson correlation coefficient and two-sided t-test p value are noted. Source data for all plots are provided as source data files**.**

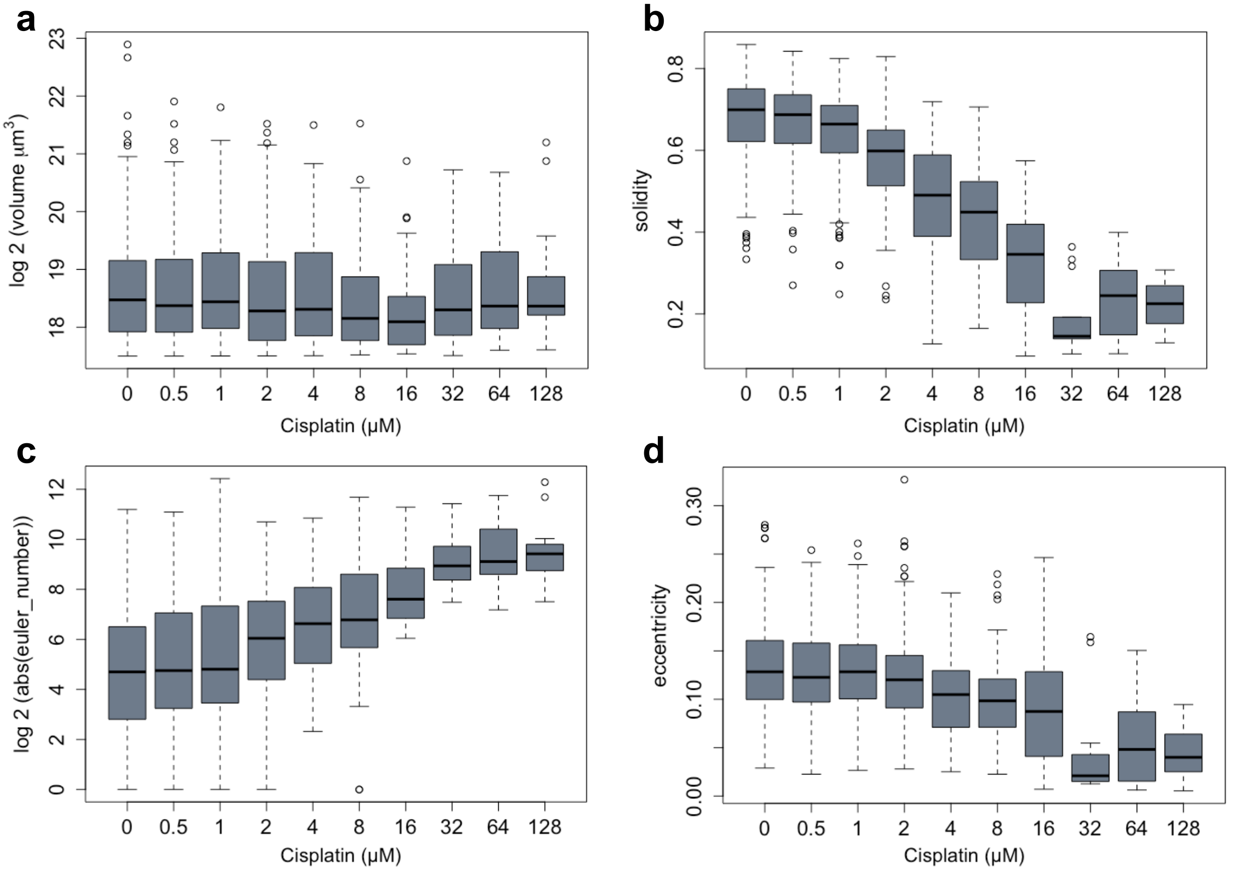

Supplemental Figure 11: Changes in organoid morphologies due to cisplatin treatment. **a.** Volume , **b.** solidity, **c.** Euler number and **d.** eccentricity for segmented large organoids after exposure to range of cisplatin treatment for 4 days (n= 1077 organoids). Median values for each condition is shown by horizontal line in the boxplot, the box denotes the IQR, the whiskers extend to a maximum of 1.5 times the IQR and outliers are shown as dots. Source data for all plots are provided as source data files**.**

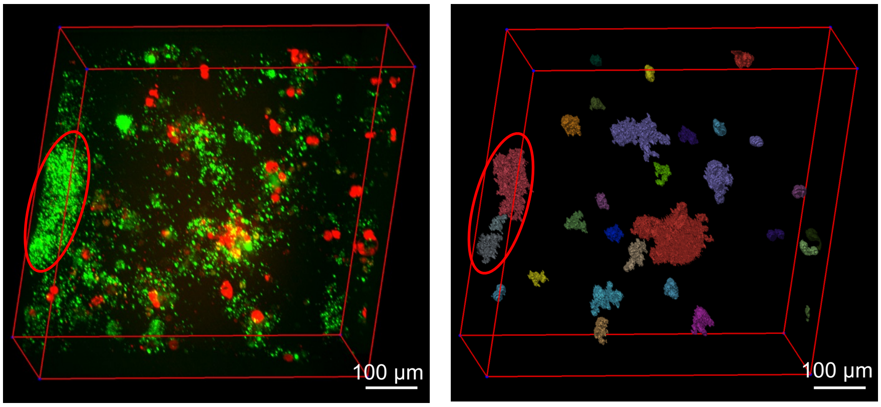

Supplementary Figure 12: Constraints of organoid segmentation in conditions with extreme cell death. Image showing example of organoid segmentation (right panel) limitation after exposure to high concentration of drug (Cisplatin =128µM, left panel). Circled region shows debris segmented as organoids

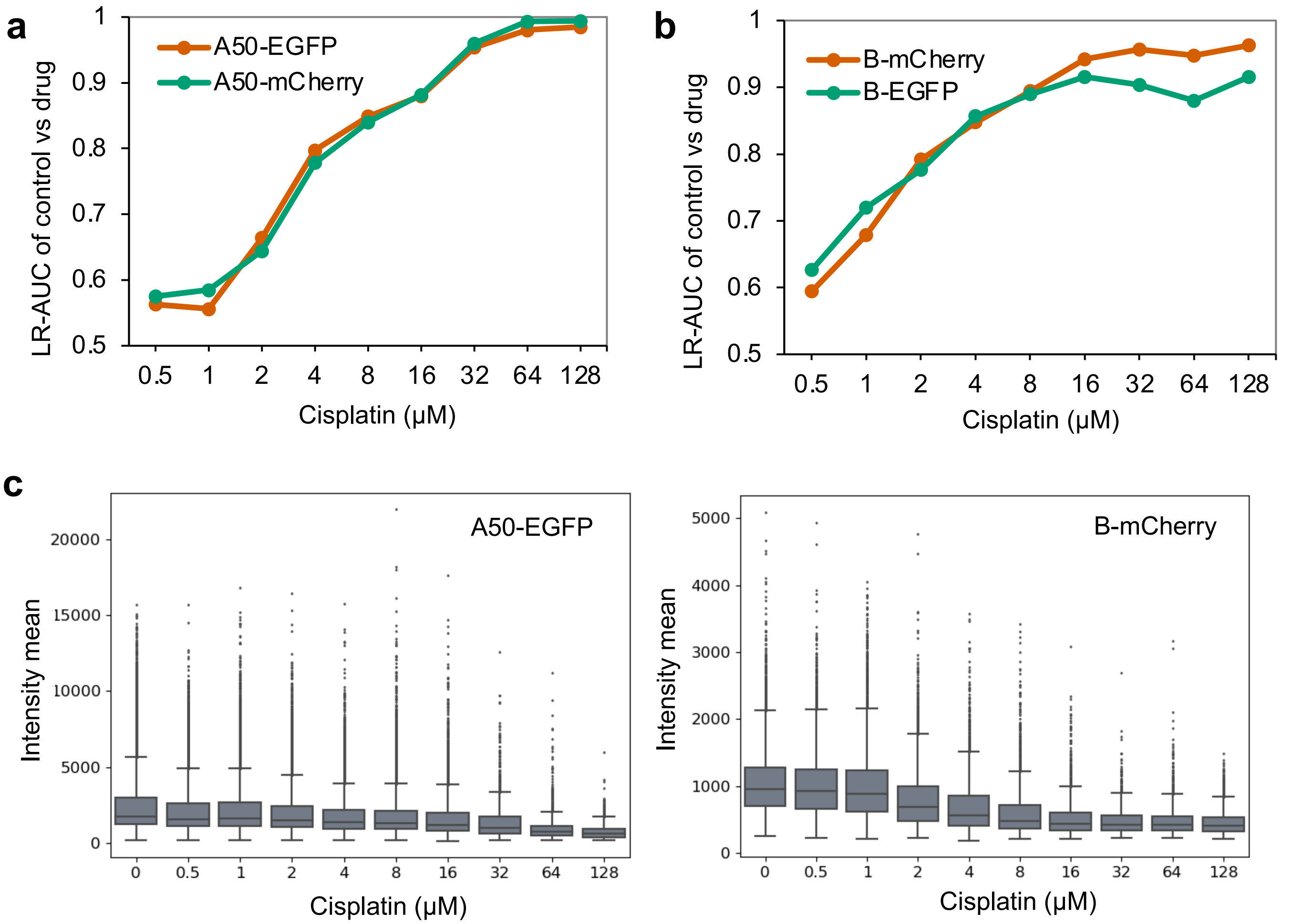

Supplementary Figure 13: Changes in nuclear morphologies due to cisplatin treatment. a. Logistic regression classifications AUC of nuclear morphologies of A50-EGFP or A50-mCherry cells when comparing nuclei in control with nuclei exposed to cisplatin for 4 days. **b**. Logistic regression classifications AUC of nuclear morphologies of B-EGFP or B-mCherry cells when comparing nuclei in control with nuclei exposed to cisplatin for 4 days. 771,571 nuclei in total were analyzed for **a** and **b**. **c.** Mean intensity of A50-EGFP (left, n= 85,494 nuclei) and B-mCherry nuclei (right, n= 51,010 nuclei) after cisplatin exposure for 4 days. Horizontal line in the boxplot indicates the median, the box denotes the IQR , the whiskers extend to a maximum of 1.5 times the IQR and outliers are shown as dots. Source data for all plots are provided as source data files**.**

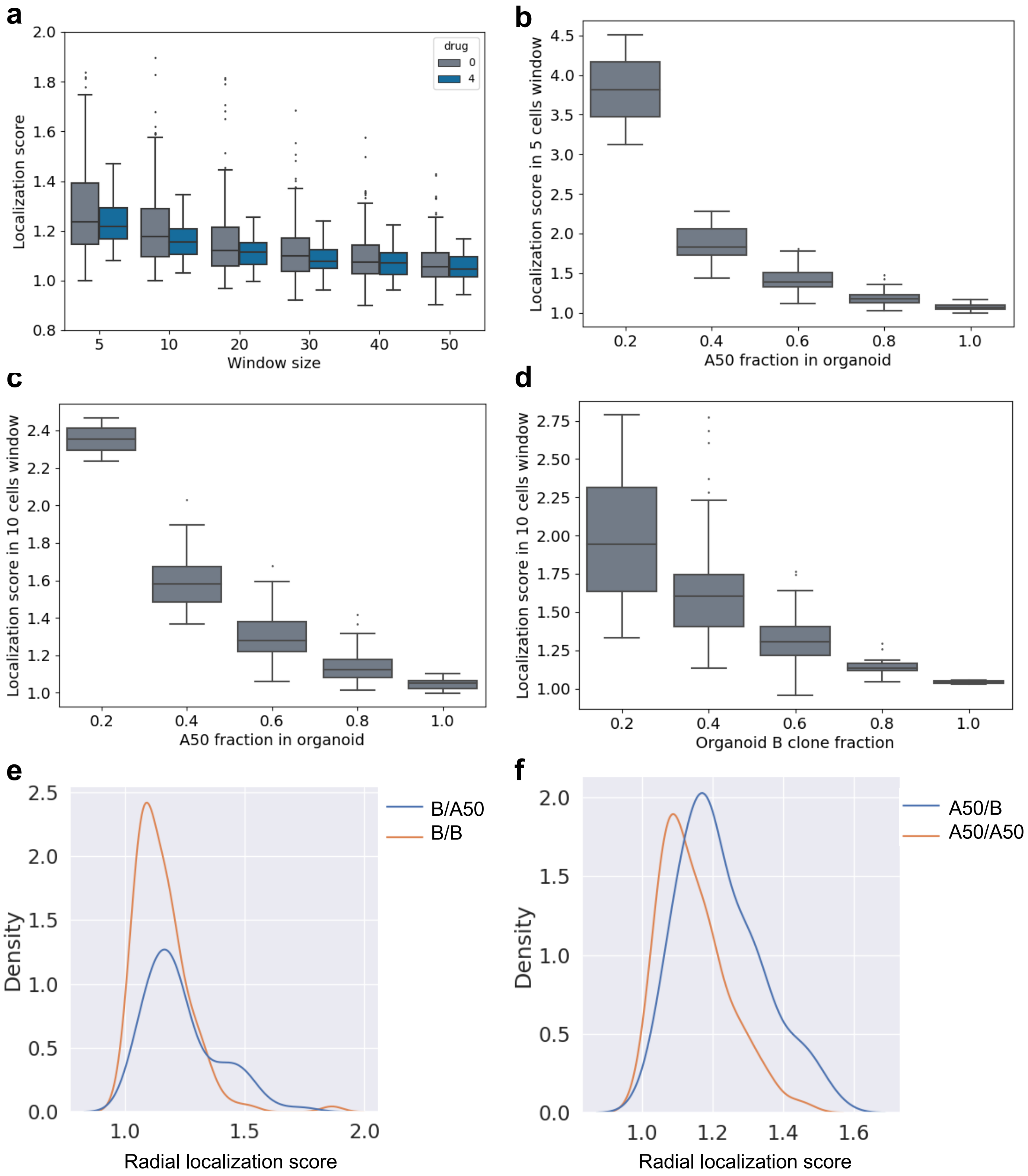

Supplementary Figure 14: Identification of cellular localization in organoids by Cellos. a. Distribution of localization score vs. window size (5, 10, 20, 30, 40, 50) for A50-EGFP cells within heterogeneously mixed organoids. Organoids from control and 4 μM cisplatin exposure for 4 days were used for this analysis (n= 274 organoids). **b, c** Localization scores for A50-EGFP cells in the 5 and 10 cell

window size respectively in organoids with varying proportions of A50 clone fraction are plotted. Organoids are grouped in bins with increments of 0.2 for the A50 cell fraction. (n= 235 organoids). **d**. Localization scores for B-mCherry cells (10 cell window size) in organoids with varying proportions of B clone fraction are plotted (n= 232 organoids). For boxplots in **a-d**, median values for each condition is shown by horizontal line in the boxplot, the box denotes the IQR , the whiskers extend to a maximum of 1.5 times the IQR and outliers are shown as dots. **e**. Distribution of radial localization score of B clones when mixed with A50 (blue line) or with alternately labeled B (orange line) (n= 152 organoids). **f.** Distribution of radial localization score localization score of A50 clones when mixed with B (blue line) or with alternately labeled A50 (orange line) (n= 212 organoids). Source data for all plots are provided as source data files**.**

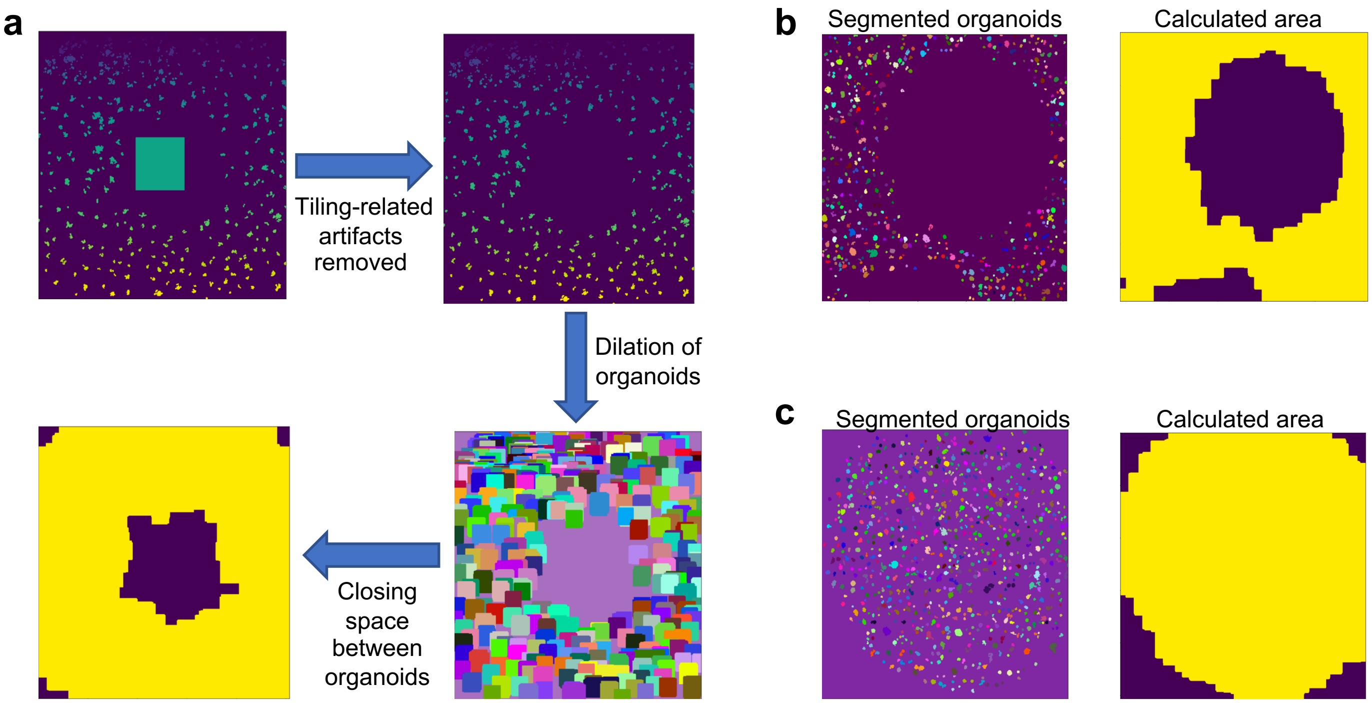

Supplementary Figure 15: Computational process of calculating image area that contains organoids. a. Schematic representation of the pipeline to detect the area of the image with organoids in focus. This area detected is highlighted in yellow in bottom left panel. **b, c.** Examples of segmented organoids image (left panel) and Cellos-detected area of the image with organoids in focus, shown in yellow (right panel). Images are z-axis maximum projections.

	Day 0				Day 4			
	A50		B		A50		B	
Seeded EGFP(%)	EGFP(%)	STDV	EGFP(%)	STDV	EGFP(%)	STDV	EGFP(%)	STDV
20	25.251	0.456	21.255	0.982	24.867	0.4	22.41	0.426
40	42.556	1.167	38.366	1.493	40.608	0.852	37.535	0.915
60	59.928	0.494	55.458	1.143	54.924	0.845	52.194	1.348
80	76.829	0.376	74.597	0.489	71.054	0.145	70.138	1.392

Supplementary Table 1: Detection of multiple fluorescently labelled cell populations in homogeneously mixed organoids. EGFP and mCherry labeled cells were mixed to form homogeneously mixed organoids of A50 or B clones. Pre-determined EGFP percentages of 20, 40, 60 and 80 were used and organoids were imaged at day 0 and day 4. Table showing percentage of EGFP labeled cells, and standard deviation (STDV) of three replicate wells for each condition. 1,123,444 cells were evaluated for this analysis .

Pipeline	Multiple organoids per well	3D/2D segmentation
CALYPSO [1]	Yes	2D
OrganoSeg [2]	Yes	2D
OrgaQuant [3]	Yes	2D
Larsen, B.M., et al. [4]	Yes	2D
Spiller, E.R., et al. [5]	Yes	2D
MOrgAna [6]	No	2D
Hof, L., et al. [7]	Yes	2D
OrganoID [8]	Yes	2D
Phindr3D [9]	Yes	2D
Zhang, L., et al. [10]	**Yes**	**3D**
Beghin, A., et al. [11]	No	3D
Boutin, M.E., et al. [12]	No	3D
**Cellos**	**Yes**	**3D**

Supplementary Table 2: Comparison of Cellos and other organoids segmentation techniques.

SUPPLEMENTARY REFERENCES:

[1] Bulin, A.L., Broekgaarden, M. and Hasan, T., Comprehensive high-throughput image analysis for therapeutic efficacy of architecturally complex heterotypic organoids. *Scientific reports*, *7*(1), p.16645 (2017).

[2] Borten, M.A., Bajikar, S.S., Sasaki, N., Clevers, H. and Janes, K.A. Automated brightfield morphometry of 3D organoid populations by OrganoSeg. *Scientific reports*, *8*(1), p.5319. (2018).

[3] Kassis, T., Hernandez-Gordillo, V., Langer, R. and Griffith, L.G. OrgaQuant: human intestinal organoid localization and quantification using deep convolutional neural networks. *Scientific reports*, *9*(1), p.12479 (2019).

[4] Larsen, B.M., Kannan, M., Langer, L.F., Leibowitz, B.D., Bentaieb, A., Cancino, A., Dolgalev, I., Drummond, B.E., Dry, J.R., Ho, C.S. and Khullar, G. A pan-cancer organoid platform for precision medicine. *Cell reports*, *36*(4) (2021)

[5] Spiller, E.R., Ung, N., Kim, S., Patsch, K., Lau, R., Strelez, C., Doshi, C., Choung, S., Choi, B., Juarez Rosales, E.F. and Lenz, H.J. Imaging-based machine learning analysis of patient-derived tumor organoid drug response. *Frontiers in oncology*, *11*, p.771173 (2021).

[6] Gritti, N., Lim, J.L., Anlaş, K., Pandya, M., Aalderink, G., Martínez-Ara, G. and Trivedi, V. MOrgAna: accessible quantitative analysis of organoids with machine learning. *Development*, *148*(18), p.dev199611 (2021).

[7] Hof, L., Moreth, T., Koch, M., Liebisch, T., Kurtz, M., Tarnick, J., Lissek, S.M., Verstegen, M.M., van der Laan, L.J., Huch, M. and Matthäus, F. Long-term live imaging and multiscale analysis identify heterogeneity and core principles of epithelial organoid morphogenesis. *BMC biology*, *19*, pp.1-22 (2021).

[8] Matthews, J.M., Schuster, B., Kashaf, S.S., Liu, P., Ben-Yishay, R., Ishay-Ronen, D., Izumchenko, E., Shen, L., Weber, C.R., Bielski, M. and Kupfer, S.S. OrganoID: A versatile deep learning platform for tracking and analysis of single-organoid dynamics. *PLoS computational biology*, *18*(11), p.e1010584 (2022).

[9] Mergenthaler, P., Hariharan, S., Pemberton, J.M., Lourenco, C., Penn, L.Z. and Andrews, D.W. Rapid 3D phenotypic analysis of neurons and organoids using data-driven cell segmentation-free machine learning. *PLOS Computational Biology*, *17*(2), p.e1008630 (2021).

[10] Zhang, L., Wang, L., Yang, S., He, K., Bao, D. and Xu, M. Quantifying the drug response of patient-derived organoid clusters by aggregated morphological indicators with multi-parameters based on optical coherence tomography. *Biomedical Optics Express*, *14*(4), pp.1703-1717 (2023).

[11] Beghin, A., Grenci, G., Sahni, G., Guo, S., Rajendiran, H., Delaire, T., Mohamad Raffi, S.B., Blanc, D., de Mets, R., Ong, H.T. and Galindo, X. Automated high-speed 3D imaging of organoid cultures with multi-scale phenotypic quantification. *Nature Methods*, *19*(7), pp.881-892 (2022).

[12] Boutin, M.E., Voss, T.C., Titus, S.A., Cruz-Gutierrez, K., Michael, S. and Ferrer, M. A high-throughput imaging and nuclear segmentation analysis protocol for cleared 3D culture models. *Scientific reports*, *8*(1), p.11135 (2018).
